# Telmisartan and Insulin Resistance in HIV (TAILoR): protocol for a dose-ranging phase II randomised open-labelled trial of telmisartan as a strategy for the reduction of insulin resistance in HIV-positive individuals on combination antiretroviral therapy

**DOI:** 10.1136/bmjopen-2015-009566

**Published:** 2015-10-15

**Authors:** Sudeep P Pushpakom, Claire Taylor, Ruwanthi Kolamunnage-Dona, Catherine Spowart, Jiten Vora, Marta García-Fiñana, Graham J Kemp, John Whitehead, Thomas Jaki, Saye Khoo, Paula Williamson, Munir Pirmohamed

**Affiliations:** 1 Department of Molecular and Clinical Pharmacology, The Wolfson Centre for Personalised Medicine, University of Liverpool, Liverpool, UK; 2 MRC Centre for Drug Safety Science, University of Liverpool, Liverpool, UK; 3 Department of Molecular and Clinical Pharmacology, University of Liverpool, Liverpool, UK; 4 Clinical Trials Research Centre, University of Liverpool, Liverpool, UK; 5 Department of Biostatistics, University of Liverpool, Liverpool, UK; 6 Department of Diabetes and Endocrinology, The Royal Liverpool and Broadgreen University Hospitals NHS Trust, Liverpool, UK; 7 Magnetic Resonance and Image Analysis Research Centre, University of Liverpool, Liverpool, UK; 8 Department of Mathematics and Statistics, Lancaster University, Lancaster, UK

## Abstract

**Introduction:**

Telmisartan, an angiotensin receptor blocker, has beneficial effects on insulin resistance and cardiovascular health in non-HIV populations. This trial will evaluate whether telmisartan can reduce insulin resistance in HIV-positive individuals on combination antiretroviral therapy.

**Methods and analysis:**

This is a phase II, multicentre, randomised, open-labelled, dose-ranging trial of telmisartan in 336 HIV-positive individuals over a period of 48 weeks. The trial will use an adaptive design to inform the optimal dose of telmisartan. Patients will be randomised initially 1:1:1:1 to receive one of the three doses of telmisartan (20, 40 and 80 mg) or no intervention (control). An interim analysis will be performed when half of the planned maximum of 336 patients have been followed up for at least 24 weeks. The second stage of the study will depend on the results of interim analysis. The primary outcome measure is a reduction in insulin resistance (as measured by Homeostatic Model Assessment—Insulin Resistance (HOMA-IR)) in telmisartan treated arm(s) after 24 weeks of treatment in comparison with the non-intervention arm. The secondary outcome measures include changes in lipid profile; body fat redistribution (as measured by MRI); plasma and urinary levels of various biomarkers of cardiometabolic and renal health at 12, 24 and 48 weeks. Serious adverse events will be compared between different telmisartan treated dose arm(s) and the control arm.

**Ethics and dissemination:**

The study, this protocol and related documents have been approved by the National Research Ethics Service Committee North West—Liverpool Central (Ref: 12/NW/0214). On successful completion, study data will be shared with academic collaborators. The findings from TAILoR will be disseminated through peer-reviewed publications, at scientific conferences, the media and through patient and public involvement.

**Trial registration numbers:**

04196/0024/001-0001; EUDRACT: 2012-000935-18; ISRCTN: 51069819.

**Strengths and limitations of this study**

- This clinical trial will evaluate whether telmisartan can reduce insulin resistance in HIV-positive individuals on combination antiretroviral therapy; this may lead to the repositioning of telmisartan to treat metabolic disease.
- The trial will use an adaptive design to inform the optimal dose of telmisartan for reduction of insulin resistance. This design also allows stopping of the trial midway if none of the doses show a statistically significant effect after the interim analysis, thereby reducing the duration of trial and related costs.
- The trial is assessing a surrogate marker (insulin resistance) as an outcome measure in this trial. Despite the fact that there is a good relationship between insulin resistance and cardiovascular health, this represents a limitation of the trial.

## Background and rationale

Combination antiretroviral therapy (cART) is the mainstay for treatment of HIV and has dramatically improved the morbidity and mortality associated with HIV, turning it into a chronic disease. However, cART, together with the virus itself, can result in various metabolic complications, including metabolic syndrome, type 2 diabetes (T2DM) and an increased risk of cardiovascular disease (CVD).^1^ These metabolic complications associated with cART also occur with HIV lipodystrophy (also known as the fat redistribution syndrome), a clustering of morphological and metabolic abnormalities comprising peripheral fat loss (lipoatrophy), visceral lipid hypertrophy, insulin resistance and dyslipidaemia,^2^ which also increases the risk of CVD.^3^

The prevalence of metabolic syndrome is high in cART treated HIV-infected patients (ranges from 11.2–45.4% in different HIV populations);^4^ the HIV DAD cohort (n=33 347) found the prevalence of metabolic syndrome to increase from 19.4% to 41.6% over a 6-year period with patients having metabolic syndrome showing a fourfold increase in the incidence of T2DM and a twofold to threefold increased risk of developing CVD.^5^ These results have been confirmed by the Multicenter AIDS Cohort Study (n=1278)^6^ and a more recent analysis of the DAD cohort.^7^ Cumulative exposure to cART also results in an increased risk of myocardial infarction with both protease inhibitors^8^ (PIs) and nucleoside reverse transcriptase inhibitors^9^ (NRTIs), as well as in intima-media thickness and an increase in the prevalence of carotid lesions.^10^

Insulin resistance, a key feature of HIV lipodystrophy and metabolic syndrome, has been described as central to cardiometabolic disease and is considered to be an important link between features of metabolic syndrome, obesity, dyslipidaemia, T2DM and CVD.^11^ In vitro studies^12^ and single drug studies in healthy individuals^13^ and HIV-infected patients^14^ ^15^ have shown that PIs and NRTIs cause insulin resistance. The prevalence of insulin resistance in cART-treated HIV-infected patients ranges from 10 to 37%,^14–16^ indicating a significant role for cART in its development. Several mechanisms have been suggested to be responsible for cART-induced insulin resistance; these include cART-induced inhibition of adipocyte differentiation,^17^ increased secretion of adipokines such as interleukin 6 (IL-6) and tumour necrosis factor α (TNF-α),^18^ and impairment of the insulin signalling pathway.^12^

Clinical intervention to arrest or reverse cART-associated insulin resistance has been suggested as a strategy to reduce the incidence of T2DM and CVD in HIV-positive patients. Insulin sensitisers such as thiazolidinediones and metformin have been trialled but results from randomised clinical trials in HIV patients have shown mixed results.^19^ ^20^ Moreover, the associated adverse effects may limit their use in HIV-infected patients.^21^ ^22^ Therefore, there is a need for novel clinical interventions with proven safety profile that can reduce cART-induced insulin resistance in HIV-infected individuals.

Some angiotensin receptor blockers (ARBs) have a beneficial effect on insulin resistance and T2DM, owing to their action on the renin-angiotensin system and partial agonist activity at PPARγ, an important regulator of adipocyte function. Telmisartan shows maximal potency on PPARγ when compared to other ARBs and has been reported to reduce insulin resistance in several in vitro*,*^23^ ^24^ animal^25^ ^26^ and clinical studies.^27–30^ Telmisartan also improves adiponectin levels, an important metabolic marker of insulin resistance and atherosclerotic disease, lipid control, and has favourable effects on fasting serum insulin and high sensitivity C reactive protein^27^ (high-sensitivity C reactive protein (hs-CRP); a marker of CVD). Telmisartan has also been shown to reduce visceral, but not subcutaneous, fat accumulation in patients with metabolic syndrome.^31^ ^32^ Importantly, telmisartan already has a license for cardiovascular protection in a broad group of at-risk patients (ONTARGET trial; 120 000 patient-years of follow-up).^33^

In contrast to the non-HIV population, the effect of telmisartan on insulin resistance in cART-treated HIV-positive patients has not been assessed. Using in vitro adipocyte models, we (Pushpakom, unpublished) and others^34^ have shown that telmisartan partially reverses the antiadipogenic effects of antiretrovirals. Our trial has therefore been designed to address this. Furthermore, although the dose-response relationship of telmisartan in hypertension is well known, whether this would also be similar in reducing insulin resistance is unclear. Our in vitro study in fact suggested that there might be a non-monotone (bell shaped) relationship of telmisartan on markers of adipocyte health. We have therefore utilised an adaptive trial design during the initial stage of the study to carefully assess the dose–response relationship of telmisartan in vivo.

## Objectives

The primary objective of the trial is to determine the effect of telmisartan on insulin resistance in HIV-positive individuals on cART using Homeostatic Model Assessment—Insulin Resistance (HOMA-IR). HOMA-IR is a measurable, validated surrogate marker of insulin resistance.^35^

The secondary objectives include assessing the optimal dose of telmisartan that can significantly reduce insulin resistance, evaluation of tolerability of telmisartan in HIV patients and mechanistic evaluation of the metabolic effects of telmisartan. The mechanistic evaluation of telmisartan will explore longitudinal changes in plasma markers that are important indicators of cardiometabolic health (adiponectin, IL-6, resistin, TNFα, hs-CRP and lipids) at different time points; it will also utilise MRI and ^1^H MR spectroscopy (MRS) to assess the effect of telmisartan on total body fat and intrahepatic and intramyocellular triglyceride content, respectively. The MRI/MRS evaluation will be limited to a subset of participants who are recruited locally.

Telmisartan is known to possess renoprotective effects;^36^ ^37^ in addition to the above objectives, the study will also assess its effects on the kidney using urinary markers of renal injury (conventional markers such as creatinine, urea, total protein and novel biomarkers such as KIM-1, NGAL and RBP).

## Trial design

This study is a phase II, multicentre, randomised, open-labelled, dose-ranging trial of telmisartan in HIV-positive individuals over a period of 48 weeks. The sample size for the study is 336 (see Sample size calculation), but a total of 370 patients will be recruited to participate in this study to account for patient withdrawals (estimated to be 10%).

The optimal dose of telmisartan that elicits the desired response is not known; hence, an adaptive design is utilised for this study. The first stage of the study will be dose-ranging where patients will be randomised 1:1:1:1 to receive one of three doses of telmisartan (20, 40 and 80 mg) or no intervention (control). An interim analysis will be performed when half of the planned maximum of 336 patients have been followed up for at least 24 weeks. The second stage of the study will depend on the results of interim analysis, which could be one of the three outcomes listed below:

1. One or more active dose groups are substantially more effective than control; this will lead to a stopping of the study and the corresponding dose(s) will be taken directly into phase III.
2. No dose shows sufficient promise at the interim analysis; this will also lead to a stopping of the study.
3. At least one of the doses shows some improvement over control at interim analysis; this will lead to a second stage where that/these dose(s) will be followed up along with the control for a further 24 weeks (total: 48 weeks). Additional patients will also be recruited to these dose(s) and control. If at the final analysis a large enough reduction in 24 week HOMA-IR score is found, the corresponding active dose will be recommended for phase III.

**Figure 1:**
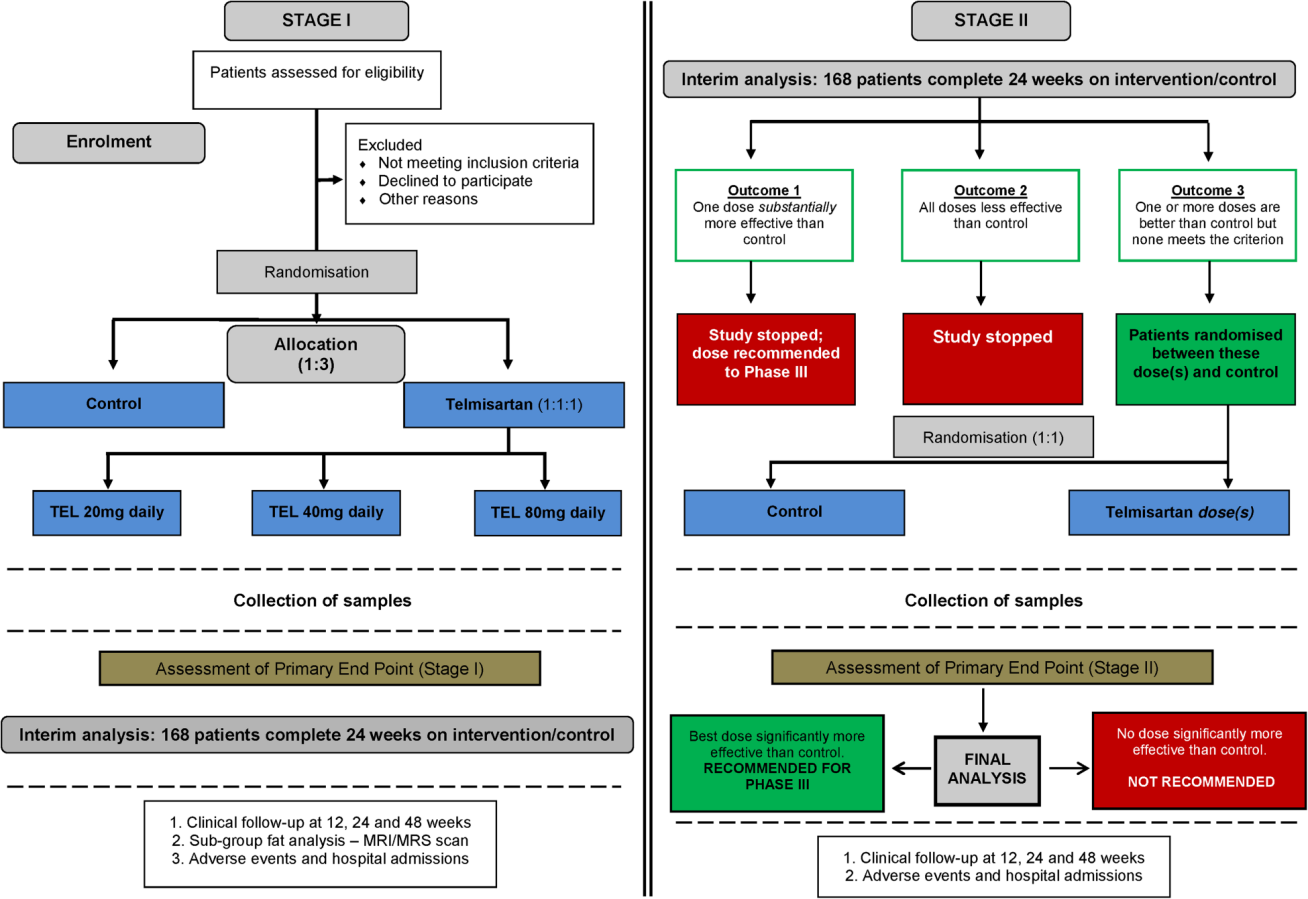
Flow Diagram for TAILoR Trial. The trial will be conducted in two stages. Stage 1 of the study is dose-ranging and patients will be randomised 1:1:1:1 to receive one of three doses of telmisartan or no intervention (control). An interim analysis will be performed when half of the planned maximum of 336 patients have been followed up for at least 24 weeks. Stage II of the study will depend on the results of interim analysis, which could be one of the three outcomes shown in the figure. All assessments will be carried out at baseline and at weeks 12, 24 and 48 post-treatment with telmisartan, as well as in the control arm. For those who participate in the MRI/MRS substudy, assessment will be at baseline and 24 weeks. TEL: Telmisartan.

In the telmisartan 40 and 80 mg arms, dose titration will be undertaken over 2−4 weeks in order to step up to the allocated dose (as per the Summary of Product Characteristics; SPC), or else the maximum tolerated dose if the target is not achieved. All assessments will be carried out at baseline and at weeks 12, 24 and 48 post-treatment with telmisartan, as well as in the control arm. For those who participate in the MRI/MRS substudy, assessment will be at baseline and 24 weeks. The study flow diagram is given in figure 1.

The Clinical Trials Research Centre (CTRC), University of Liverpool, is the coordinating centre for this study (http://www.liv.ac.uk/translational-medicine/research/ctrc/about/).

## Patient recruitment

### Identification of eligible patients

Patients who are eligible for inclusion into the trial will be identified and recruited through HIV specialty centres located in the UK that have agreed to participate in the study. These HIV specialty centres are part of the secondary care system and are mostly based in an urban setting. Participants will be identified by the clinical team at each centre via a search of the patient database(s) either electronically or manually or a clinic list review to find potentially eligible patients. The inclusion and exclusion criteria are detailed in box 1.

Box 1 **Inclusion and exclusion criteria**

*Inclusion criteria*

1. Adult (age 18 or above) HIV-positive individuals receiving antiretroviral therapy for at least 6 months. The antiretroviral therapy may contain:
  - A boosted protease inhibitor (LPV/r, ATV/r, DRV/r, FAPV/r, SQV/r)
  - and/or efavirenz, rilpivirine or etravirine
    The backbone can be based on N(t)RTI, raltegravir or maraviroc. Patients on protease inhibitor monotherapy will be included if they meet other criteria. Patients on nevirapine or dolutegravir regimens, without concomitant boosted PIs, should not be included. Additionally, patients on elvitegravir, which is administered in combination with cobicistat (as Stribild), should not be recruited.
2. Ability to give informed consent
3. Willingness to comply with all study requirements

*Exclusion criteria*

1. Pre-existing diagnosis of type 1 or 2 diabetes (Fasting glucose >7.2 mmol/L or HbA1c≥6.5% [48 mmol/mol] or abnormal OGTT or random plasma glucose≥11 mmol/L)
2. Patients known to have consistently low blood pressure (pre-existing hypotension; A reading below a threshold of 100/60 mm Hg on three separate occasions)
3. Patients with renal disease (eGFR<60 in the 6 months preceding randomisation)
4. Patients with known untreated renal artery stenosis
5. Patients with cholestasis, biliary obstructive disorders or severe hepatic impairment.
6. Patients with evidence of an active, chronic hepatitis C infection
7. Patients who are on unboosted ATV
8. Patients who are on/ have been on hormone therapy, anabolics and insulin sensitisers within 6 months preceding randomisation. Patients on hormonal contraception are eligible.
9. Patients who are already on/ have been on other ARBs, ACE inhibitors or direct renin inhibitors within 4 weeks preceding randomisation.
10. Those with suspected poor compliance
11. Pregnant or lactating women
12. Women of childbearing age unless using reliable contraception, for example, coil, barrier method, hormonal contraceptive that does not interact with their antiretroviral therapy
13. Co-enrolment in other drug trials
14. Patients who have participated in a trial of an IMP likely to influence insulin sensitivity, plasma insulin, glucose levels or plasma lipid levels within 6 months preceding randomisation.
15. For the subcohort of patients undergoing MRI/MRS, normal MR exclusion criteria will apply (See Body fat distribution substudy).

ARB, angiotensin receptor blockers; ATV, atazanavir; DRV, darunavir; eGFR, estimated glomerular filtration rate; FAPV, fosamprenavir; HbA1c, glycated haemoglobin; IMP, investigational medicinal product; LPV/r, lopinavir/ritonavir; N(t)RTI, nucleoside (nucleotide) reverse transcriptase inhibitors; OGTT, oral glucose tolerance test; PI, protease inhibitor; SQV, saquinavir.

### Consent procedure

At the routine clinic visit, eligible patients are informed of the study by a member of the clinical team or research staff. A Patient Information Sheet and instructions on how to proceed if they are interested in taking part will be provided by the research nurse. All patients will be provided with a full explanation of the trial and given sufficient time to consider their decision before obtaining informed written consent. In consenting to the trial, patients are consenting to trial treatment, follow-up and data collection. Patients are free to withdraw consent at any time without providing a reason. Follow-up of these patients will be continued through the trial research nurses and the lead investigator at each centre unless the participant explicitly also withdraws consent for follow-up.

### Baseline assessments

Once informed consent has been obtained from the patient, they will be booked in for a baseline assessment visit within 30 days of giving consent. The patient will be advised to arrive fasting when reporting for the baseline assessment. The research team will conduct the baseline assessments and complete the eligibility and baseline case report form (CRF) during the baseline assessment visit. The baseline assessments include fulfilment of eligibility criteria; recording demographic details; full medical and drug history; body weight and vital signs; and waist/thigh circumference. A urine pregnancy test is offered for females of childbearing potential since telmisartan is not recommended during the first trimester of pregnancy and is contraindicated during the second and third trimesters of pregnancy due to its teratogenic potential. However, a refusal to undertake a pregnancy test will not preclude trial entry. Blood samples (for plasma, serum and DNA) and urine will also be collected from each patient at the time of baseline screening. Table 1 details the schedule of study assessments conducted.

**Table 1:** Schedule of study procedures

			T+2 week	T+4 weeks				
Time	Pre T0 At each recruitment site	T0 Randomisation/baseline*	Dose titration—40/80 mg arms (dose given 40 mg)	Dose titration for 80 mg arm (dose given 80 mg)	T+12 weeks Follow-up	T+24 weeks Follow-up	T+48 weeks End of treatment	Premature withdrawal of consent
Database search to identify potential participants or clinic list review	Х							
Information sheet provided to patient	X							
Signed informed consent		Х						
Assessment of eligibility criteria by a medically qualified person		Х						
Review of medical history (including collection of most recent blood test results for urea and electrolytes, eGFR, liver function, diabetes screening, etc		Х†					X	Х
Review of concomitant medications		Х	Х	Х	Х	Х	Х	Х
Urine pregnancy test		Х			X	X		
Randomisation		Х						
Study intervention		Х	Х	Х	Х	Х		
Compliance with study intervention—patient diaries and pill counting			X	X	Х	Х	Х	
Physical examination—complete		Х						
Physical examination—symptom-directed			Х	Х	Х	Х	X	Х
Height		Х						
Weight		Х			Х	Х	Х	Х
Waist/thigh circumference		Х			Х	Х	Х	Х
Heart rate, blood pressure		Х	Х	Х	Х	Х	Х	Х
Collection of 3 fasting blood samples for bioanalysis		Х			Х	Х	Х	Х
Collection of urine sample		X			X	X	X	X
Assessment of adverse events			Х	Х	Х	Х	Х	Х
Consent for substudy		X						
MRI/MRS scan for substudy		X				X		

(X)—As indicated/appropriate.

*Baseline assessment and randomisation visit should be within 30 days of the patient giving consent.

†Liver function and diabetes screening result only to be collected at baseline.

eGFR, estimated glomerular filtration rate; MRS, MR spectroscopy.

## Randomisation

Participants will be randomised to receive telmisartan 20, 40, 80 or control (no intervention) in a 1:1:1:1 ratio once (A) eligibility criteria have been fulfilled; (B) fully informed written consent has been obtained; and (C) baseline assessments have been completed. Participants will be randomised using a bespoke secure (24 h) web based randomisation programme controlled centrally by the CTRC, University of Liverpool. For each recruiting centre, randomisation will be stratified by ethnicity (African-American and non-African-American) where ethnicity is determined by self-categorisation using the NHS ethnicity codes. Centres will be provided with emergency back-up randomisation envelopes to be used in the event of a system failure or when a system failure cannot be resolved in a reasonable time frame. Patients may only be randomised into the study by an authorised member of staff at the study site as detailed on the delegation log. Participants may only be randomised into the study once.

## Trial interventions

Telmisartan is an angiotensin receptor antagonist indicated for clinical use as an antihypertensive agent. It is also used to reduce cardiovascular events in patients who are at risk. However, the current trial uses telmisartan outside its licensed indications.

At the onset of the trial, telmisartan was under patent (Boehringer Ingelheim GmBH; Micardis); however, during the course of the trial, the patent expired and several manufacturers started marketing generic telmisartan, which was then also used in the trial, but the trial will continue to use Micardis SPC as the reference SPC. Telmisartan used in this trial is sourced via usual local NHS procurement arrangements.

Telmisartan tablets are available in 20, 40 or 80 mg doses and therefore fit in with the dose range to be used in the trial prior to interim analysis. In most cases, the participant is provided their required dose in one tablet. Telmisartan tablets are for once-daily oral administration and should be taken with liquid, with or without food. The CRFs will be used to record which brand has been dispensed to the participant. Telmisartan is stored as per the manufacturer's SPC.

For each randomised patient, treatment is for a maximum period of 48 weeks. The principal investigator or delegated other will issue a prescription based on the patient's randomisation status and the trial treatment can start immediately after randomisation. For the three treatment arms, treatments will be dispensed at the appropriate doses at baseline, at 12 weeks and then at 24 weeks, unless interruption or discontinuation is warranted. Wherever titration of dose is required, the treatment starts with 20 mg and is then titrated upwards over a period of 2 weeks (for a 40 mg dose) or 4 weeks (for an 80 mg dose). At 48 weeks, administration of trial treatments will be stopped and any unused medications will be returned to pharmacy for disposal via their local procedures. There is a 2 week attendance window on either side of each of the follow-up visits and a 4 day window on either side of the titration visits.

Since the results of the interim analysis decide the design of stage II of the trial, those patients who are on a dose that is not taken forward to stage II will be asked to stop taking the medication completely. These patients will continue to be monitored for any adverse events for a period of 7 days (washout period for telmisartan) after which they will no longer be part of the trial and will return to routine care. For those who are on trial arms whose dose(s) are taken forward to stage II, they will be asked to continue on the same dose for a further 24 weeks. Since treatment is not stopped between stages I and II, some participants may receive up to a maximum of 48 weeks trial treatment before the results of the interim analysis are known. For patients recruited after the results of interim analysis are known, they will be randomised equally to the non-intervention (control) arm and the remaining telmisartan dose arm(s).

Dose modifications will be allowed in those who are randomised to a particular dose arm but do not tolerate that dose. The patient will be allowed to continue on the nearest dose tolerated. Those who show adverse effects as a result of the trial intervention or due to the HIV therapy may be withdrawn from the trial treatment. The decision to interrupt or discontinue trial therapy is at the discretion of the treating physician using their informed clinical opinion. Any changes will be documented in the CRF along with the justification for those changes. Patients who withdraw from study treatment will be asked to allow continuation of scheduled evaluations, complete an end-of-study evaluation if appropriate and be given appropriate care under medical supervision until the symptoms of any adverse event resolve or the patient's condition becomes stable. Follow-up of these patients will be continued through the trial research nurses and the lead investigator at each centre unless the participant explicitly also withdraws consent for follow-up. Data up to the time of withdrawal will be included in the analyses unless the patient explicitly states that this is not their wish.

## Patient follow-up

Apart from the dose-titration visits for participants in the 40 and 80 mg arms, follow-up visits will be designed to fit with routine hospital visits where possible. The study will also allow a 2 week window on either side of the scheduled follow-up visit date to ensure flexibility. Individual patients will be sent reminders about follow-up visits by the research nurses provided they have agreed to receive them. If any of the trial patients are lost to follow-up, contact will be attempted through the research nurse and lead investigator at each centre. Wherever possible, information on the reason for loss to follow-up will be recorded.

## Outcomes and assessments

Efficacy of trial treatments will be assessed throughout the period of the study. The primary outcome measure is a reduction in insulin resistance (as measured by HOMA-IR) in telmisartan treated arm(s) after 24 weeks of treatment in comparison with the non-intervention arm. Fasting plasma and serum samples will be collected from each participant at baseline and at follow-up visits (weeks 12, 24 and 48) and stored under appropriate conditions locally. Biochemical analyses will be carried out centrally in an accredited clinical laboratory. Fasting plasma glucose will be measured by standard clinical methods and serum insulin will be measured by an electrochemiluminescence immunoassay using a Cobas C Analyser (Roche Diagnostics, Switzerland). HOMA-IR will be calculated using the equation: (fasting serum insulin (mU/L)×fasting plasma glucose (mmol/L))/22.5. The secondary outcome measures are detailed in box 2. Serum and urine biomarker analyses and DNA extraction will be performed centrally using Human Multiplex ELISA (Millipore) on a BioPlex 200 System (BioRad) and Chemagic Magnetic Separation Module I (MSM I), respectively.

Box 2 **Secondary outcome measures**

1. Change in lipid profile (total cholesterol, triglycerides, LDL-c and HDL-c) at weeks 12, 24 and 48 between telmisartan treated arm(s) and the control arm.
2. Change in body fat redistribution as measured by MRI/MRS at 24 weeks between telmisartan treated arm(s) and control arm (See substudy).
3. Change in plasma concentrations of biomarkers (adiponectin, lipin1, IL-6, TNF-α, resistin and hs-CRP) at 12, 24 and 48 weeks between telmisartan treated arm(s) and control arm.
4. Change in insulin resistance, measured longitudinally at weeks 12 and 48, in telmisartan treated arm(s) in comparison with the control arm.
5. Change in urinary biomarker levels at 12, 24 and 48 weeks between telmisartan treated arm(s) and the control arm.
6. Difference in expected and unexpected serious adverse events between different telmisartan treated dose arm(s) and the control arm over the study follow-up.

HDL-c, high density lipoprotein-cholesterol; hs-CRP, high sensitive, C reactive protein; IL-6, interleukin-6; LDL-c, low density lipoprotein-cholesterol; TNF-α, tumour necrosis factor-α.

### Assessment of compliance with study treatment/s

All participants on intervention arms are given a treatment diary to record their daily treatment compliance. Compliance with the study treatment will be ascertained on the basis of what is recorded in the treatment diary and by recording the number of pills remaining in the packs.

### Body fat redistribution substudy

A substudy will be undertaken only for patients recruited from the North West of UK to assess whether telmisartan results in any changes in the total body adipose content and intrahepatic and intramyocellular lipid content. This will be assessed at baseline and at 24 weeks by MRI and ^1^H MRS in an on-site MRI research facility. Patients recruited will be given a separate patient information sheet and consent form containing information on the substudy and requirements for MRI/MRS. Participants will be allowed to withdraw from the substudy anytime but remain in the main study. Only patients who consent to take part in the main study and satisfy the normal MR exclusion criteria (normal MR exclusion criteria include patients using pacemakers, cochlear implants, piercings, metal in the head or elsewhere in the body, and those who suffer from claustrophobia) will be included in the substudy. MRI of the total body adipose content will be undertaken on a Siemens 1.5 T Symphony scanner (Siemens, Erlangen Germany) using well-established methods.^38^ MRI will be analysed to obtain volume estimates of total body subcutaneous, total internal, subcutaneous abdominal and intra-abdominal adipose tissue. In the same subcohort of patients, liver and skeletal muscle ^1^H MR spectra will be acquired using the Siemens body coil and Siemens CP extremity coil, respectively, using established methods.^39^ Analysis of all imaging data will be conducted centrally.

## Sample size calculation

The primary response from each patient is the difference between the baseline HOMA-IR score and their HOMA-IR score at 24 weeks. The design has been constructed under the assumption that for all patients this response is normally distributed with a common SD, σ.

The sample size calculation is based on a one-sided type I error of 5% and a power of 90%. If there is no difference between the mean response on any treatment and that on control, then a probability of 0.05 is set for the risk of erroneously ending the study with a recommendation that any treatment be tested further. For the power, we adopt a generalisation of this power requirement to multiple active treatments due to Dunnett.^40^ Effect sizes are specified as the percentage chance of a patient on active treatment achieving a greater reduction in HOMA-IR score than a patient on control as this specification does not require knowledge of the common SD, σ. The requirement is that, if a patient on the best active dose has a 65% chance of a better response than a patient on control, while patients on the other two active treatments have a 55% chance of showing a better response than a patient on control, then the best active dose should be recommended for further testing with probability 1−β=0.90. A 55% chance of achieving a better response on active dose relative to control corresponds to a reduction in mean HOMA-IR score of about a sixth of an SD (0.178σ), while the clinically relevant effect of 65% corresponds to a reduction of about half an SD (0.545σ). The critical values for recommending that a treatment is taken to further testing at the interim and final analyses (2.782 and 2.086) have been chosen to guarantee these properties using a method described by Magirr *et al*,^41^ generalising the approach of Whitehead and Jaki.^42^

The maximum sample size of this study is 336 evaluable patients, although the use of the interim analysis may change the required sample size. The study will recruit additional patients to account for an anticipated 10% dropout rate.

### Interim monitoring and analyses

An interim analysis will take place once the primary end point is available for at least 42 patients on each arm (ie, half of the planned maximum of 336 patients). The sample SD pooled across all four arms is used to construct test statistics expressing the advantage of each of the three active treatments over control. The analysis will be proceeding as follows:

1. If the largest test statistic exceeds 2.782, the study will be stopped and the corresponding dose will be recommended for further testing.
2. If any active dose shows no improvement over control (ie, has a negative test statistic), that active dose will be dropped.
3. If no active dose shows an improvement over control, the study will be stopped and no significant improvement over control will be claimed.
4. If some improvement over control is detected for at least one dose (ie, at least one test statistic is between 0 and 2.782), the study will progress to the second stage.

At the interim analysis, doses may be dropped from the trial, or the trial may be stopped altogether. Consequently, the required sample size when the decision is reached could be smaller than the maximum stated number of 336 patients. The values 168 (if the study is stopped following interim analysis), 252 (if one active dose arm is promoted to the second stage), 294 (if two active dose arms are promoted to the second stage) and 336 (if all three active dose arms are promoted to the second stage) are possible. The reduced sample sizes refer to the numbers of patients with 24 week HOMA-IR scores which are included in the analysis. Evaluation of the patient withdrawal rate will be carried out and the sample size will be adjusted accordingly. There will be additional patients who have been recruited during the 24 weeks prior to extracting the data for interim analysis and their number will depend on the recruitment rate achieved. A decision to discontinue recruitment, in all patients or in selected subgroups, will be made on the basis of results from the interim analysis by the Independent Data and Safety Monitoring Committee (IDSMC).

## Statistical analysis

### Primary outcome analysis

Three different doses of the intervention will be evaluated against the control in stage 1 of the study and an interim analysis will take place that will allow ineffective doses to be eliminated quickly while a dose showing a positive effect can be taken forward. An ANCOVA model will be fitted for a 24 week HOMA-IR score adjusting for the stratification factor (African-American and non-African-American), and the corresponding t—values will be used to construct test statistics expressing the advantage of each of the active treatments over control. The largest of these test statistics will be compared to the interim critical value (2.782) and proceed as discussed above at the interim analysis. At the final analysis, if the largest comparative test statistic exceeds the final critical value (2.086), then this dose would be recommended for further study. Adjustments can be made to allow for any discrepancies between target and actual sample sizes while still preserving the one-sided type I error rate at 0.05.

### Secondary outcome analysis

Linear mixed effect models will be used to analyse secondary and mechanistic outcomes. The evaluation of beneficial and adverse biomarkers in relation to insulin resistance will be examined using the joint modelling approach^43^ ^44^ accounting for informative loss to follow-up or censoring. Structural equation models^45^ will be used to assess the inter-relationship between multiple biomarkers over effect of treatment while accounting for time-varying confounders. Mechanistic outcomes such as change in body fat, liver and muscle fat distribution will be analysed using a multiple linear regression model. Differences will be considered significant at p<0.05. Differences between the groups will be estimated with 95% CIs.

## Safety reporting

The CTRC will be notified of all serious adverse reactions (SAR), serious adverse events (SAE) and suspected unexpected serious adverse reactions (SUSARs) within 24 h of the local site becoming aware of the event. The CTRC will notify the MHRA and main Research Ethics Committee (REC) of all SUSARs occurring during the study on behalf of the chief investigator according to the following timelines: fatal and life-threatening within 7 days of notification and non-life threatening within 15 days. It will also submit an annual report of all SAEs to the sponsor, MHRA and the main REC and provide the IDSMC with listings of all SAEs on an ongoing basis. The study may be prematurely discontinued on the basis of new safety information, or for other reasons given by the IDSMC and/or Trial Steering Committee (TSC), sponsor or REC concerned. All investigators will be informed of all SUSARs occurring throughout the study. The assignment of the severity/grading of adverse events will be made by the investigator responsible for the care of the participant using the Division of AIDS Table for Grading the Severity of Adult and Paediatric Adverse Events V.1.0 (2009) definitions.^46^ The CTRC will monitor SAE and ADR reporting rates across sites during the course of the trial and if any inconsistencies are noted, they will be investigated and additional training provided.

### Reporting of pregnancy

Female study participants of childbearing potential will be offered a pregnancy test as part of the trial screening process and at weeks 12 and 24. Any pregnancy which occurs during the study will be reported as an SAE to the CTRC within 24 h of the site becoming aware of its occurrence and the participant will be instructed immediately to stop taking study drugs. All pregnancies that occur during treatment need to be followed up until after the outcome. The investigator will discuss the risks of continuing with the pregnancy and the possible effect to the fetus with the participant.

## Ethical considerations

The conduct of this study will be in accordance with the Declaration of Helsinki, 1964 and later revisions.

The main ethical issue is the potential allocation of participants to less effective treatment arms. In stage 1 of the trial, a quarter of the patients will be allocated to the non-intervention control arm; it is also likely that some of the intervention arms could be found to be less effective during the interim analysis and hence be dropped. However, these comparator arms are necessary for the identification of a positive drug effect in the treatment arm(s) and its optimal dose. This does not have any impact on the control of HIV infection since the intended use of telmisartan in this patient population is only as an adjuvant drug and not as the primary drug to treat HIV infection.

Telmisartan is an antihypertensive drug, and thus there is a possibility that some of the participants randomised to the higher doses may experience hypotension. The eligibility criteria aim to exclude those who consistently show hypotension; moreover, the prevalence of telmisartan-induced hypotension in normotensive individuals has been found to be rare in previous studies.^47^ ^48^ However, the trial will take adequate precautions such as routine blood pressure monitoring to address this issue. There will be a minor increase in the pill burden to the participants of this trial; however, this is not a major issue since the intervention is available as a single tablet that needs to be taken only once daily.

Other ethical issues include contraception for all women of childbearing age during the course of the trial and additional clinic visits required for baseline assessments and dose titration (limited to only 40 and 80 mg arms). For a subset of patients recruited to undertake the substudy, it may involve additional patient time to undertake MRI/MRS scans. In the event that the study is discontinued, participants will be treated according to standard clinical care.

### Ethical and regulatory approvals

The study, this protocol and related documents have been approved by the National Research Ethics Service Committee North West—Liverpool Central (Ref: 12/NW/0214).

This study falls within the remit of the EU Directive 2001/20/EC, transposed into UK law as the UK Statutory Instrument 2004 No 1031: Medicines for Human Use (Clinical Trials) Regulations 2004 as amended. This trial has been registered with the Medicines and Healthcare Products Regulatory Agency (MHRA) and granted a Clinical Trial Authorisation (04196/0024/001-0001). The EUDRACT number is 2012-000935-18.

## Data collection and trial monitoring

### Data collection

Data management procedures for the trial will be developed and overseen by the CTRC, University of Liverpool. The CTRC will provide training, essential documentation and user support to the study centres, and monitoring, if triggered by an incident or where appropriate. All primary data will be entered into the study CRF for this study. Each participant will be assigned a unique screening number at the start of the assessment, which will be recorded on the consent form and the baseline assessment CRF and on all other documents used to record participant data. All original CRFs will be returned to the CTRC. For the participant treatment diaries, the participant initials and randomisation number will be clearly labelled on all documents. The laboratory read-outs will be obtained for blood, urine samples and for the body fat distribution substudy from automated equipment. These will be uploaded securely to the central trial database.

### Trial monitoring

Trial monitoring procedures for this study are based on a risk assessment conducted by the CTRC, University of Liverpool. Guidance issued by the MRC, Department of Health and the MHRA on risk-adapted approaches to the management of CTIMPs proposes a three-level categorisation for the potential risk associated with an IMP.^49^ In this study, telmisartan is used outside the manufacturer's indication; therefore, the IMP here is categorised as *Type B:* ‘*somewhat higher than that of standard medical care*’. This level of risk will inform the risk assessment, regulatory requirements, nature and extent of the monitoring, and the management processes used in the trial.

### Central monitoring

Data stored at the CTRC will be checked for missing or unusual values and for consistency within participants over time. Any suspect data will be returned to the site in the form of data queries and sites are expected to respond to these queries with an explanation/resolution to the discrepancies. There are a number of monitoring features in place at the CTRC to ensure reliability and validity of the trial data.

### Clinical site monitoring

The CTRC personnel may need direct access to primary data such as patient records and laboratory reports; since this affects the patient's confidentiality, this fact is included on the patient information sheet. Individual participant medical information obtained as a result of this study is considered confidential and disclosure to third parties is prohibited. Medical information may be given to the participant's medical team and all appropriate medical personnel responsible for the participant's welfare. The only identifiable data transferred is the consent form and this is disclosed in the patient information sheet and consent form. The CTRC will preserve the confidentiality of participants taking part in the study and The University of Liverpool is registered as a Data Controller with the Information Commissioners Office.

### Trial management and oversight

#### Trial Management Group

The Trial Management Group (TMG) will be responsible for the day-to-day running and management of the trial and will meet, as a minimum, approximately 10 times a year. The TMG will comprise the Chief Investigator, other lead investigators (clinical and non-clinical) and members of the trial coordinating centre (CTRC).

#### Trial Steering Committee

The TSC will meet at least once annually and will provide overall supervision for the trial and provide advice through its independent Chairperson. The ultimate decision for the continuation of the trial lies with the TSC. The TSC will consist of an independent chairperson (with clinical expertise in HIV), two independent statisticians with expertise in adaptive trial design and medical statistics, a user representative, the investigators, representatives of the research networks, sponsors and principal investigators.

#### Independent Data and Safety Monitoring Committee

The IDSMC will meet at least once annually and will be responsible for reviewing and assessing recruitment, interim monitoring of safety and effectiveness, trial conduct and external data. It will provide a recommendation to the TSC concerning the continuation of the study. The IDSMC will consist of an independent chairperson (with clinical expertise in HIV) and two independent members: one who is an expert in the field of HIV lipodystrophy, and one who is an expert in medical statistics and adaptive trial design. Terms of reference for the TMG and TSC and Charter for the IDSMC are available on request from the CTRC, University of Liverpool.

## Notification of amendments

Any amendments made to the study including protocol amendments will be communicated to the appropriate agencies for approval prior to implementation. All substantial amendments made will be reviewed by the sponsor prior to submission to the REC and the MHRA for their approval; substantial amendments will also be notified to all participating research sites. All substantial and non-substantial amendments as well as respective regulatory approvals will be provided electronically via the Integrated Research Application System (IRAS) to the Clinical Research Network North West Coast who will notify the Research and Development Department at individual participating sites for local approval and implementation.

## Time frame and trial status

TAILoR is currently recruiting from 19 specialist HIV centres throughout the UK. The study has so far recruited 293 patients and has been given a no-cost extension to continue recruitment till the end of July 2015. Each participant is followed up for a total of 48 months. The total study period is 56 months.

## Discussion

Metabolic disease and insulin resistance continue to be a major problem in HIV-infected individuals; a recent longitudinal study observed an overall insulin resistance prevalence of 21% (using a HOMA-IR cut-off >3.8) in HIV patients.^50^ Given that HIV is now considered a chronic disease with an ageing population^51^ and the fact that ageing further increases the susceptibility to age-related comorbidities such as metabolic and CVD, the magnitude of this problem is likely to become even greater. This is exemplified by the fact that the prevalence of insulin resistance in HIV patients increases with age, ranging from 5% in patients <30 years to 30% in patients over 60 years of age.^50^ Therefore, there is a pressing need to develop or identify newer therapies to combat metabolic disease in this group of individuals; this trial will potentially address this need and may lead to the repositioning of telmisartan to treat metabolic disease.

Since the start of this trial, two smaller studies have already reported beneficial metabolic effects of telmisartan in cART-treated HIV patients. While one study observed a reduction in HOMA-IR with 80 mg telmisartan,^52^ the other did not find a reduction in HOMA-IR but observed a loss of total and subcutaneous fat with a 40 mg dose.^53^ This clearly underlines the need for a well-powered trial to confirm the efficacy of telmisartan for reducing insulin resistance and this is met by the current study. The trial also utilises a novel adaptive design which will enable identification of the optimal dose of telmisartan, if ultimately it is found to elicit a statistically significant beneficial effect on insulin resistance. The adaptive design also allows stopping of the trial midway if none of the doses show a statistically significant effect after the interim analysis; this will reduce the duration of the trial and result in cost saving.

Of course, we are assessing a surrogate marker (insulin resistance) as an outcome measure in this trial. Despite the fact that there is a good relationship between insulin resistance and cardiovascular health,^54^ ^55^ this represent a limitation of the trial. However, this is a phase IIb trial, and thus a surrogate marker as a primary outcome measure is justified, because a trial to show a reduction in cardiovascular end points will necessarily need to be large and would be reserved for a follow-on phase III design.
